# ADGRD1 promotes bladder cancer progression and angiogenesis via the PI3K/AKT/mTOR-mediated pro-angiogenic secretome

**DOI:** 10.3389/fonc.2026.1817872

**Published:** 2026-05-28

**Authors:** Chen Li, Zhichao Yang, Jialei Zhang, Xiaodong Yan, Hongwei Su

**Affiliations:** 1 Department of Urology, The First Affiliated Hospital of Hebei North University, Zhangjiakou, Hebei, China; 2 Department of Urology, Hebei North College, Zhangjiakou, Hebei, China

**Keywords:** ADGRD1, adhesion GPCR, angiogenesis, bladder cancer, PI3K/Akt/mTOR signaling, tumor progression

## Abstract

**Background:**

Bladder cancer (BLCA) remains a major cause of cancer-related mortality, particularly in advanced and metastatic stages. Identifying molecular drivers of tumor aggressiveness is critical for developing targeted therapies. Adhesion G protein–coupled receptor D1 (ADGRD1, also known as GPR133) is an orphan aGPCR recently implicated in tumorigenesis. However, its role in bladder cancer and underlying signaling mechanisms remain unknown.

**Methods:**

The clinical significance of ADGRD1 was analyzed using GEPIA3 datasets, focusing on clinical stage and prognosis. Stable ADGRD1-knockdown and overexpression BLCA cell lines were established. qPCR, Western blot, CCK-8, colony formation, wound-healing, Transwell, and HUVEC tube formation assays were used to assess proliferation, migration, invasion, and angiogenesis. Xenograft models were used to evaluate *in vivo* tumor growth. PI3K/AKT/mTOR pathway activity was examined by Western blot, and its function validated using SC79 (AKT activator) and LY294002 (PI3K inhibitor).

**Results:**

ADGRD1 expression was significantly enriched in advanced-stage and high-grade BLCA, where it serves as a robust predictor of poor overall survival and progression-free interval. Functional assays demonstrated that ADGRD1 promotes BLCA cell proliferation, migration, invasion, and endothelial tube formation *in vitro*, and enhances tumor growth and angiogenesis *in vivo*. Importantly, ADGRD1-high cells enhanced endothelial tube formation by upregulating a secretome rich in VEGF, PDGF, and IL-8. Mechanistically, ADGRD1 activated the PI3K/AKT/mTOR signaling cascade, as evidenced by increased phosphorylation of pathway components. Pharmacological inhibition of PI3K/AKT/mTOR reversed the oncogenic and pro-angiogenic effects of ADGRD1.

**Conclusions:**

This study identifies ADGRD1 as a key progression-associated driver in BLCA. By modulating the PI3K/AKT/mTOR signaling axis and the pro-angiogenic microenvironment, ADGRD1 facilitates tumor growth and neovascularization. ADGRD1 may serve as a promising prognostic biomarker and therapeutic target for advanced BLCA.

## Introduction

Bladder cancer (BLCA) is one of the most common malignant tumors of the urinary system, ranking tenth in global cancer incidence and representing a major cause of cancer-related mortality, particularly among men (1). According to recent epidemiological data, approximately 573,000 new BLCA cases and 213,000 deaths occur worldwide annually (2). Despite advances in surgical resection, chemotherapy, and immunotherapy, the prognosis for patients with advanced or metastatic BLCA remains poor, with a median survival of 15 months (1, 3). Tumor recurrence, invasion, and distant metastasis are major obstacles to long-term disease control. Therefore, elucidating the molecular mechanisms that drive BLCA progression and identifying novel therapeutic targets are of critical importance.

Among the numerous signaling networks involved in tumorigenesis, the PI3K/AKT/mTOR pathway plays a pivotal role in regulating cell proliferation, survival, and angiogenesis (4). Aberrant activation of this pathway has been reported in over 40% of BLCA cases and is closely associated with poor prognosis and resistance to therapy (5). Targeting PI3K/AKT/mTOR signaling has therefore become an area of active investigation. However, the upstream regulators responsible for activating this pathway in BLCA remain incompletely understood.

Recent research has highlighted the oncogenic roles of adhesion G protein–coupled receptors (aGPCRs)–a subfamily of GPCRs characterized by large extracellular adhesion domains that mediate cell–cell and cell–matrix interactions. These receptors are increasingly recognized as key modulators of tumor growth, metastasis, and vascular remodeling (6–8). Among them, ADGRD1 (also known as GPR133) is an orphan aGPCR implicated in various physiological and pathological processes, including tissue development, hypoxia response, and tumorigenesis (9, 10). ADGRD1 has been shown to promote glioblastoma proliferation under hypoxic conditions and enhance cell survival by activating cAMP-related signaling cascades (11). Nevertheless, its role in bladder cancer biology remains unexplored.

Given the established connection between GPCR signaling and PI3K/AKT/mTOR activation (12), we hypothesized that ADGRD1 may serve as an upstream regulator of this pathway in BLCA, thereby contributing to malignant phenotypes such as enhanced proliferation, invasion, and angiogenesis. To test this hypothesis, we analyzed the expression and prognostic significance of ADGRD1 in BLCA using public datasets and conducted a series of *in vitro* and *in vivo* experiments to elucidate its biological function and molecular mechanism.

Our findings reveal that ADGRD1 is upregulated in metastatic bladder cancer, correlates with advanced clinical stage and poor prognosis, and promotes tumor progression and angiogenesis by activating the PI3K/AKT/mTOR signaling cascade. These results not only identify ADGRD1 as a potential prognostic biomarker but also suggest it may represent a promising therapeutic target for patients with advanced BLCA.

## Materials and methods

### Cell lines and culture

Human bladder cancer cell lines 5637, RT112, HT1376, and T24 (Procell, Wuhan, China), UMUC1 (Mingzhoubio, Zhejiang, China), and 253JBV (JiningBio, Shanghai, China) were maintained in RPMI-1640 medium (Gibco, USA) supplemented with 10% fetal bovine serum (FBS) (Gibco, USA), 100 U/mL penicillin, and 100 μg/mL streptomycin at 37 °C in a humidified incubator with 5% CO_2_.

Human umbilical vein endothelial cells (HUVECs) were obtained from Procell (Wuhan, China), and cultured in DMEM (Gibco, USA) containing 10% FBS under the same conditions. All cell lines were authenticated by short tandem repeat (STR) profiling and routinely tested for mycoplasma contamination.

### Bioinformatics analysis

The GEPIA3 database (http://gepia3.bioinfoliu.com/) was used to analyze ADGRD1 expression and its association with clinical outcomes using the Cancer Genome Atlas (TCGA) Bladder Urothelial Carcinoma (BLCA) dataset (n=405 samples). For survival analysis, including overall survival (OS) and progression-free interval (PFI), patients were divided into high- and low-expression groups using the median expression value as the threshold. Survival analysis was performed using the Kaplan–Meier method, and the Hazard Ratio (HR) with 95% Confidence Intervals (CI) was calculated using the Cox proportional hazards model. Correlations between ADGRD1 and angiogenesis genes (CD31/PECAM1 and CD34) were evaluated using Spearmen’s correlation.

### Plasmid construction and lentiviral transduction

Short hairpin RNAs (shRNAs) targeting ADGRD1 (sh#1, sh#2) and a non-targeting control (shNC) were cloned into the pLKO.1 lentiviral vector. For overexpression, the full-length human ADGRD1 cDNA was subcloned into the pLVX-Puro vector (Clontech, USA). Lentiviruses were produced by co-transfecting HEK293T cells (Procell, China) with the target plasmid and packaging plasmids (psPAX2 and pMD2.G) using Lipofectamine 3000 (Invitrogen, USA). The shRNA sequences targeting human ADGRD1 were: sh#1 5’-GCACCGTTACTACTATGGGAT-3’; sh#2 5’-CCAGGCCAAGTGTTATGAGAA-3’.

Bladder cancer cells were infected with viral supernatants containing 8 μg/mL polybrene and selected with puromycin (2 μg/mL) for 7 days to establish stable cell lines.

### Quantitative real-time PCR

Total RNA was extracted using TRIzol reagent (Invitrogen, USA), and cDNA synthesis was performed using the PrimeScript RT reagent kit (Takara, Japan). qPCR was carried out using SYBR Green Master Mix (Applied Biosystems, USA) on a QuantStudio 6 Flex system. GAPDH served as the internal control.

Relative gene expression was calculated by the 2^-ΔΔCt^ method. Primer sequences were as follows: ADGRD1, forward 5′-GAACTTCAGGATACAACTGGAGA-3′, reverse 5′-TTGTCCCGCGTATACAGCTC-3′. GAPDH, forward 5′-AATGACCCCTTCATTGAC -3′, reverse 5′-TCCACGACGTACTCAGCGC-3′.

### Western blot analysis

Cells or tumor tissues were lysed using RIPA buffer containing protease and phosphatase inhibitors (Beyotime, China). Equal amounts of protein (30 μg) were separated by SDS-PAGE and transferred to PVDF membranes (Millipore, USA). After blocking with 5% nonfat milk, membranes were incubated overnight at 4 °C with primary antibodies: ADGRD1 (1:1000, DF4947, Affinity Biosciences, China); PI3K (CST#4292), p-PI3K (CST#13857), AKT (CST#9272), p-AKT (CST#9271), mTOR (CST#2972), p-mTOR (CST#2971) (1:1000, Cell Signaling Technology, USA); CD31 (Cat No. 66065-2-Ig), CD34 (Cat No. 14486-1-AP), β-actin (Cat No. 66009-1-Ig) (1:2000, Proteintech, China). After incubation with HRP-conjugated secondary antibodies, proteins were visualized using ECL reagents (Thermo Fisher Scientific, USA) and quantified by ImageJ software.

### Cell proliferation assays

Cell viability was assessed using the CCK-8 assay (Beyotime, China). Cells were seeded into 96-well plates (3 × 10³ cells/well) and incubated for 0–96 h. At each time point, 10 μL of CCK-8 reagent was added, and absorbance at 450 nm was measured after 2 h.

For colony formation assays, cells (500/well) were seeded in 6-well plates and cultured for 10–14 days. Colonies were fixed with 4% paraformaldehyde, stained with 0.1% crystal violet, and counted under a microscope.

### Migration and invasion assays

For wound-healing assays, confluent monolayers were scratched with a 200 μL pipette tip and washed to remove debris. Cells were cultured in serum-free medium, and wound closure was photographed at 0 h and 24 h. Migration rate was calculated as: (initial width−final width)/initial width × 100%.

Transwell migration and invasion assays were performed using 24-well chambers with 8 μm pore filters (Corning, USA). For invasion assays, the upper chamber was precoated with Matrigel (BD Biosciences, USA). After 24 h incubation, migrated or invaded cells on the lower surface were fixed, stained with crystal violet, and counted in five random fields.

### HUVEC tube formation assay

Conditioned medium (CM) was collected from BLCA cells transfected with shNC, shADGRD1, vector, or ADGRD1-OE. HUVECs (2 × 10^-^ cells) were seeded in 96-well plates coated with Matrigel and cultured in the presence of the respective CM for 6 h. Tube-like structures were imaged using an inverted microscope (Leica, Germany). The branch number was quantified using ImageJ.

### Immunofluorescence staining

HUVECs were fixed in 4% paraformaldehyde, permeabilized with 0.1% Triton X-100, blocked with 5% BSA, and incubated with primary antibodies against CD31 (AB222783, Abcam, UK) and CD34 (AB198395, Abcam, UK) overnight at 4 °C, followed by Alexa Fluor-conjugated secondary antibodies. Nuclei were counterstained with DAPI. Fluorescence images were captured using a confocal microscope (Zeiss LSM880, Germany).

### Xenograft tumor model

Animal experiments were approved by the Laboratory Animal Ethics Committee of Lai’an Technology Co., LTD (approval number: G2025088) and were carried out in accordance with the ARRIVE guidelines regarding the care and use of laboratory animals. BALB/c nude mice (4–6 weeks old, female, n = 3 per group) were subcutaneously injected with 2 × 10^6^ BLCA cells (253JBV-shNC/shADGRD1 or 5637-Vec/OE) into the flank region. Tumor volume was measured every 3 days using the formula: V = L × W^2/^2. After 30 days, mice were euthanized, and tumors were excised, weighed, and subjected to WB and immunofluorescence staining analyses. To ensure unbiased results, mice were randomly assigned to experimental groups using a computer-generated random number sequence. During the study, researchers responsible for tumor volume measurements and final weight assessments were blinded to the group allocation.

### Pharmacological modulation of PI3K/AKT/mTOR signaling

To assess pathway dependency, SC79 (AKT activator, 10 μM, Cat No. HY-18749, MCE, USA) or LY294002 (PI3K inhibitor, 20 μM, Cat No. HY-10108, MCE, USA) was added to cells for 24 h before functional assays. Effects on proliferation, migration, invasion, and angiogenesis were evaluated using CCK-8, colony formation, wound-healing, Transwell, and tube formation assays, respectively.

### Statistical analysis

All experiments were performed in at least three independent biological replicates, and data are presented as mean ± SD. Statistical analyses were conducted using GraphPad Prism 10.1.2. Comparisons between two groups were made using two-tailed Student’s t-test, and multiple group comparisons were analyzed using one-way ANOVA with Tukey’s *post-hoc* test. Survival data were analyzed by Kaplan–Meier method and log-rank test. A P value < 0.05 was considered statistically significant.

## Results

### ADGRD1 expression is elevated in bladder cancer and correlates with poor prognosis

**Figure 1:**
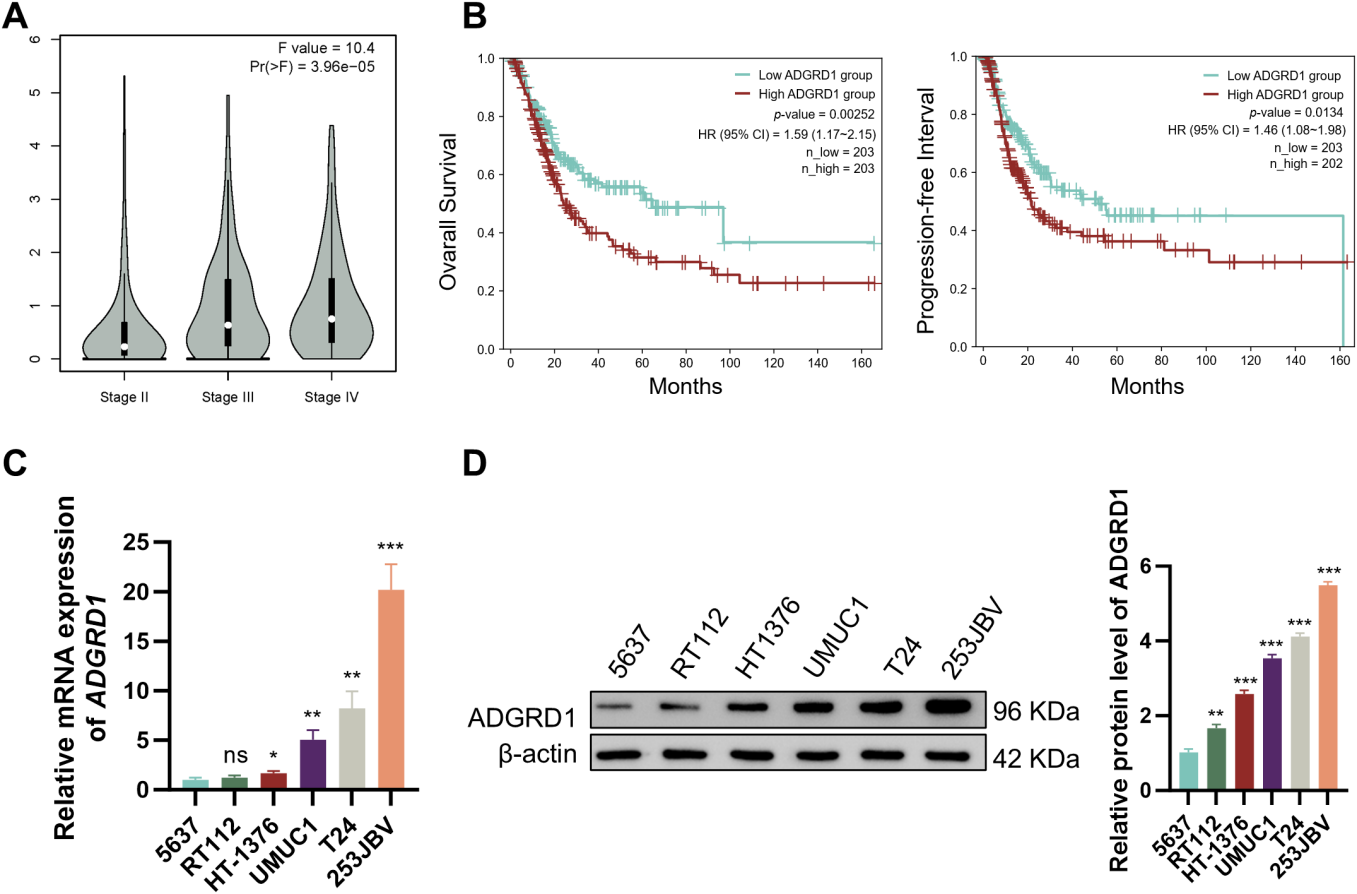
ADGRD1 is upregulated in bladder cancer and correlates with poor prognosis. **(A)** GEPIA database analysis showing increased ADGRD1 expression in bladder cancer (BLCA) tissues with advancing tumor stage (Stage II–IV). **(B)** Kaplan–Meier survival curves from GEPIA3 demonstrating that high ADGRD1 expression is associated with reduced overall survival (OS) and progression-free interval (PFI). **(C)** qPCR analysis of ADGRD1 mRNA levels in non-invasive (5637, RT112, HT1376) and metastatic (UMUC1, T24, 253JBV) BLCA cell lines. **(D)** Western blot showing higher ADGRD1 protein expression in metastatic BLCA cells compared with non-invasive counterparts. Data are presented as mean ± SD of three independent biological replicates. *P < 0.05, **P < 0.01, ***P < 0.001 vs 5637.

To provide a comprehensive evaluation of ADGRD1 expression in BLCA, we performed cross-validation using the TCGA-BLCA cohort and several GEO datasets (GSE7476, GSE40355, GSE121711, and GSE293398) (**Supplementary Figure 1**). While ADGRD1 mRNA levels showed heterogeneity and were relatively lower in tumor tissues compared to normal urothelium in some cohorts, further stratification revealed that ADGRD1 expression positively correlates with clinical advancement (**Figure 1A**). Specifically, ADGRD1 levels were significantly higher in advanced-stage (Stage III/IV) and high-grade tumors compared to their early-stage and low-grade counterparts (P<0.05; **Figure 1A**). Furthermore, high ADGRD1 expression was significantly associated with a higher risk of mortality and recurrence. Specifically, BLCA patients with high ADGRD1 expression exhibited significantly shorter overall survival (HR = 1.59, 95% CI: 1.17–2.15, P = 0.00252) and a reduced progression-free interval (HR = 1.46, 95% CI: 1.08–1.98, P = 0.0134) (**Figure 1B**).

Quantitative PCR (qPCR) confirmed that ADGRD1 mRNA levels were markedly higher in metastatic bladder cancer cell lines (UMUC1, T24, 253JBV) compared with non-invasive cell lines (5637, RT112, HT1376) (P<0.05; **Figure 1C**). Consistently, Western blot (WB) analysis demonstrated elevated ADGRD1 protein expression in metastatic cells relative to non-invasive counterparts (P<0.05; **Figure 1D**). These data indicate that ADGRD1 expression positively correlates with tumor aggressiveness and poor clinical outcomes in BLCA.

### ADGRD1 promotes proliferation, migration, and invasion of bladder cancer cells

**Figure 2:**
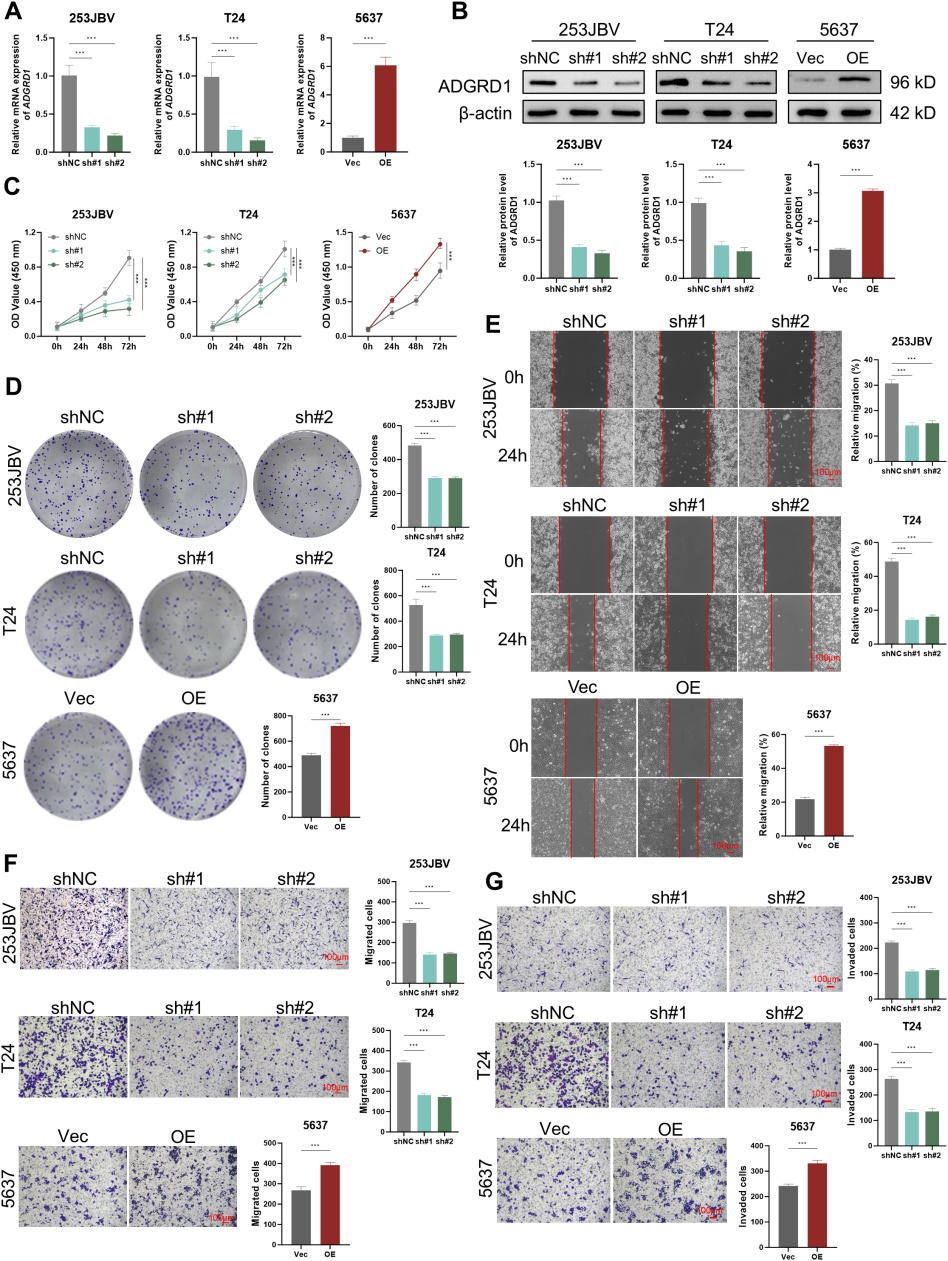
ADGRD1 promotes proliferation, migration, and invasion of bladder cancer cells *in vitro*. **(A, B)** Validation of ADGRD1 knockdown (sh#1, sh#2) in 253JBV and T24 cells and overexpression (OE) in 5637 cells by qPCR and Western blot. **(C)** CCK-8 assay showing decreased cell viability following ADGRD1 knockdown and increased viability upon ADGRD1 overexpression. **(D)** Colony formation assay confirming that ADGRD1 depletion inhibits, while overexpression enhances, clonogenic growth. **(E)** Wound-healing assay showing impaired migration in ADGRD1-silenced cells and accelerated wound closure in ADGRD1-overexpressing cells. **(F)** Transwell migration assay and **(G)** Transwell invasion assay demonstrating consistent results. Data are presented as mean ± SD of three independent biological replicates. ***P < 0.001.

To explore the biological function of ADGRD1 in BLCA, we generated cell lines with ADGRD1 knockdown (sh#1, sh#2) in high-expressing 253JBV and T24 cells, and ADGRD1 overexpression (OE) in low-expressing 5637 cells. Both qPCR and WB confirmed successful modulation of ADGRD1 expression (**Figures 2A, B**).

Functionally, CCK-8 assays showed that ADGRD1 knockdown significantly inhibited cell viability in 253JBV and T24 cells, whereas ADGRD1 overexpression enhanced 5637 cell viability (P<0.05; **Figure 2C**). Colony formation assays demonstrated that ADGRD1 depletion reduced, while its overexpression promoted, the clonogenic capacity of BLCA cells (**Figure 2D**).

Similarly, wound healing and Transwell assays revealed that ADGRD1 knockdown suppressed, while overexpression enhanced, BLCA cell migration and invasion (**Figures 2E–G**). Collectively, these results demonstrate that ADGRD1 facilitates BLCA cell proliferation, migration, and invasion *in vitro*.

### ADGRD1 enhances angiogenesis through paracrine effects on endothelial cells

**Figure 3:**
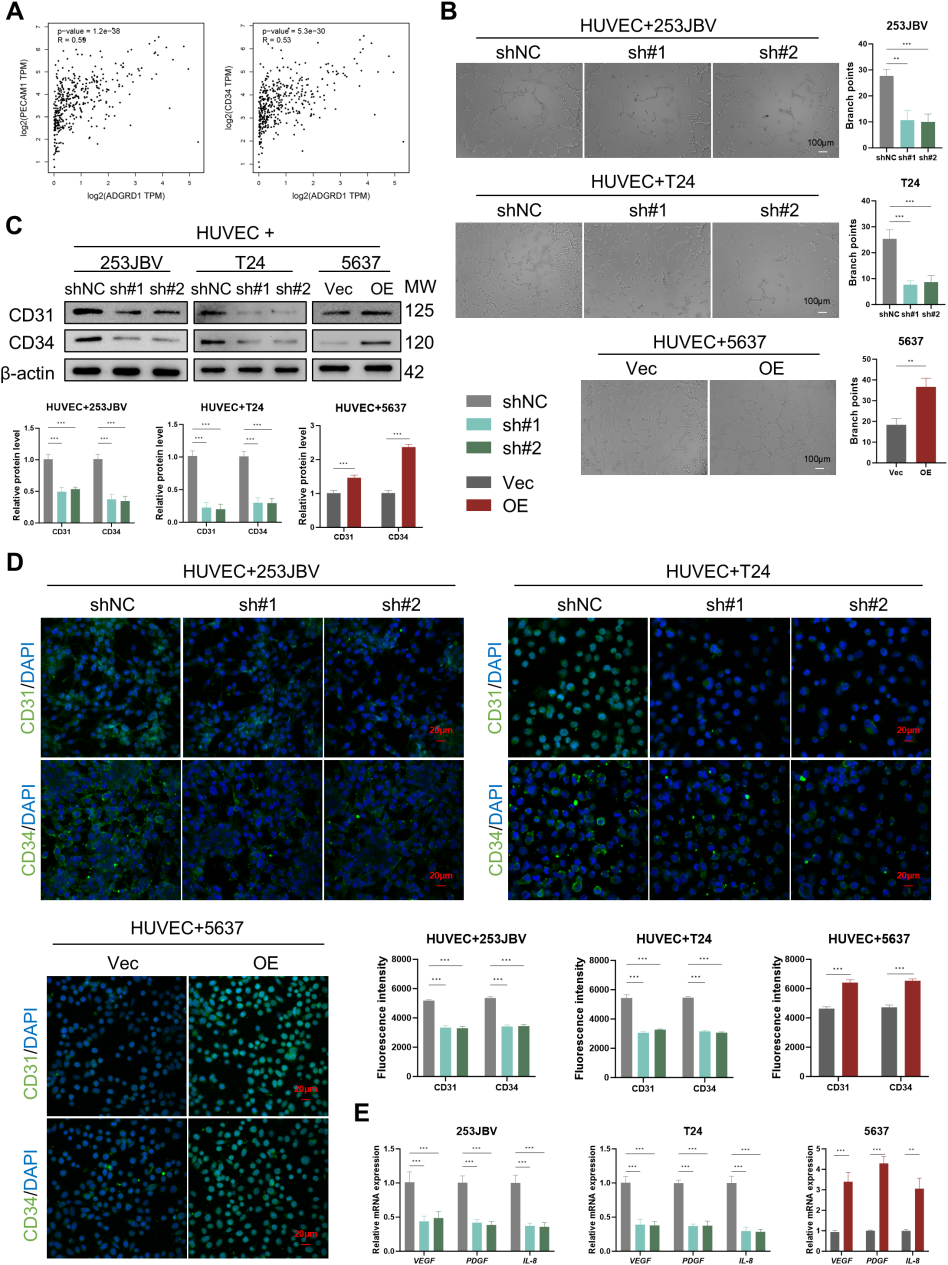
ADGRD1 enhances angiogenesis through paracrine effects on endothelial cells. **(A)** Correlation analysis in GEPIA showing positive associations between ADGRD1 and angiogenesis markers CD31 (PECAM1) and CD34 in BLCA tissues (R = 0.59, 0.53; P < 0.05). **(B)** Representative images of HUVEC tube formation after co-culture with ADGRD1-manipulated BLCA cells. Tube formation was reduced in ADGRD1-silenced (sh#1, sh#2) cells and increased in ADGRD1-overexpressing cells. Quantification of branch number is shown. Scale bars, 100 µm. **(C)** Western blot analysis of CD31 and CD34 expression in HUVECs co-cultured with indicated BLCA cells. **(D)** Immunofluorescence staining confirming consistent changes in CD31 and CD34 expression. Scale bars, 20 µm. **(E)** qPCR analysis showing the mRNA expression levels of pro-angiogenic mediators (VEGF, PDGF, and IL-8) in BLCA cells following ADGRD1 knockdown or overexpression. Data are presented as mean ± SD of three independent biological replicates. **P < 0.01, **P < 0.001.

GEPIA analysis showed that ADGRD1 expression was positively correlated with endothelial markers CD31 (PECAM1) and CD34 in BLCA tissues (R = 0.59 and R = 0.53, P<0.05; **Figure 3A**). To functionally assess this relationship, HUVEC cells were treated with conditioned medium (CM) derived from ADGRD1-manipulated BLCA cells. Microscopic observation and quantification of branch number revealed that HUVEC tube formation was markedly reduced when cultured with CM from ADGRD1-silenced 253JBV or T24 cells, but enhanced when cultured with CM from ADGRD1-overexpressing 5637 cells (**Figure 3B**). Consistent with these observations, Western blot and immunofluorescence staining showed reduced expression of CD31 and CD34 in HUVECs co-cultured with ADGRD1-silenced cells, while ADGRD1 overexpression induced the opposite effect (**Figures 3C, D**).

To identify the paracrine mediators driving these endothelial changes, we analyzed the expression of key pro-angiogenic factors in ADGRD1-modified BLCA cells. qPCR analysis demonstrated that the mRNA levels of VEGF, PDGF, and IL-8 were significantly downregulated following ADGRD1 knockdown in 253JBV and T24 cells (**Figure 3E**). Conversely, overexpression of ADGRD1 in 5637 cells led to a robust upregulation of these three mediators (**Figure 3E**). These findings indicate that ADGRD1 promotes angiogenesis in the tumor microenvironment by regulating the transcription and secretion of a potent cocktail of angiogenic cytokines.

### ADGRD1 accelerates tumor growth and angiogenesis *in vivo*

**Figure 4:**
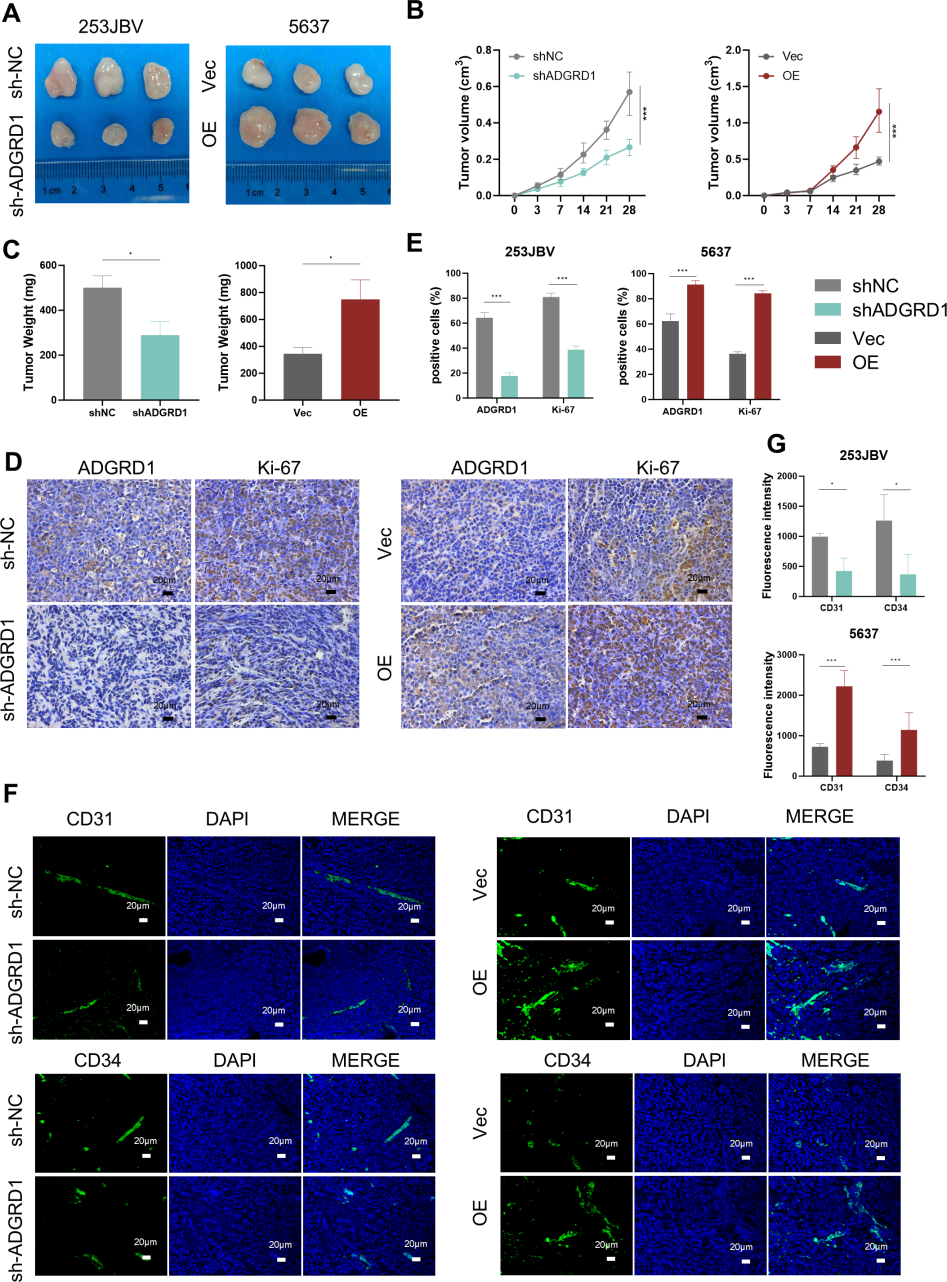
ADGRD1 promotes bladder tumor growth and angiogenesis *in vivo*. **(A)** Representative xenograft tumors derived from ADGRD1-knockdown 253JBV and ADGRD1-overexpressing 5637 cells. **(B)** Tumor growth curves showing that ADGRD1 knockdown suppressed, whereas overexpression promoted, tumor growth. **(C)** Final tumor weights measured at Day 30. **(D, E)** Representative IHC images **(D)** and quantification **(E)** of ADGRD1 and Ki-67 expression in xenograft tissues. Scale bars, 20 µm. **(F, G)** Representative Immunofluorescence images **(F)** and quantification **(G)** of angiogenic markers CD31 and CD34 demonstrating reduced microvessel density in ADGRD1-silenced tumors and increased vascularization in ADGRD1-overexpressing tumors. Scale bars, 20 µm. Data are presented as mean ± SD. N = 3 mice per group. *P < 0.05, **P < 0.001.

To evaluate the oncogenic role of ADGRD1 *in vivo* using both loss-of-function and gain-of-function approaches, xenograft mouse models were established. We utilized 253BJV cells (high baseline ADGRD1) for knockdown validation and 5637 cells (low baseline ADGRD1) for overexpression validation. Tumors derived from ADGRD1-knockdown 253JBV cells were smaller, whereas those derived from ADGRD1-overexpressing 5637 cells were larger than their respective controls (**Figure 4A**). Consistent results were obtained in tumor growth curves and final tumor weights, where ADGRD1 knockdown suppressed, and its overexpression enhanced, tumor growth (P<0.05; **Figures 4B, C**). IHC staining demonstrated that ADGRD1 protein levels were successfully suppressed in tumors derived from 253JBV-shADGRD1 cells and elevated in 5637-OE tumors compared to their respective controls (**Figures 4D, E**). Critically, we observed that the expression of Ki-67, a core marker of cellular proliferation, was significantly downregulated in the ADGRD1-knockdown group and markedly upregulated in the ADGRD1-overexpression group (**Figures 4D, E**).

Immunofluorescence staining showed decreased CD31 and CD34 expression in tumors from the ADGRD1-silenced group, while the overexpression group exhibited the opposite trend (**Figures 4F, G**). These results confirm that ADGRD1 promotes BLCA progression and angiogenesis *in vivo*.

### ADGRD1 activates the PI3K/AKT/mTOR signaling pathway

**Figure 5:**
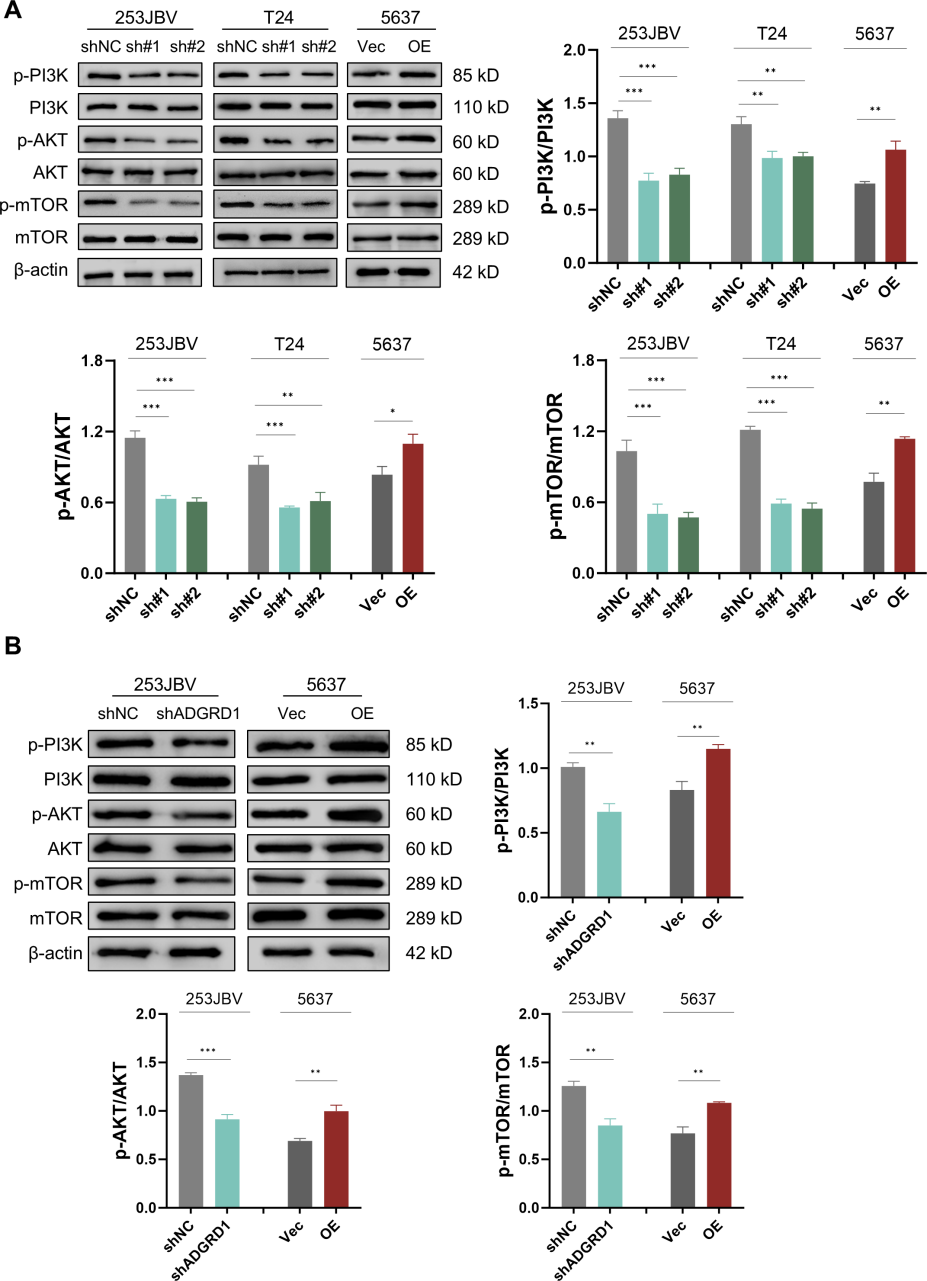
ADGRD1 activates the PI3K/AKT/mTOR signaling pathway. **(A)** Western blot showing reduced phosphorylation of PI3K, AKT, and mTOR in ADGRD1-silenced 253JBV and T24 cells, and elevated phosphorylation in ADGRD1-overexpressing 5637 cells. **(B)** Immunoblotting of xenograft tumor tissues confirming consistent regulation of PI3K/AKT/mTOR phosphorylation *in vivo*. β-actin served as the loading control. Data are presented as mean ± SD. N = 3 independent biological replicates or 3 mice per group. *P < 0.05, **P < 0.01, ***P < 0.001.

Given that the PI3K/AKT/mTOR pathway is central to tumor growth and angiogenesis, we examined whether ADGRD1 influences this signaling axis. *In vitro* WB assays revealed that ADGRD1 knockdown suppressed the phosphorylation levels of PI3K, AKT, and mTOR in 253JBV and T24 cells, while ADGRD1 overexpression enhanced their phosphorylation in 5637 cells (**Figure 5A**).

Similarly, tumor tissue lysates from xenograft models showed that ADGRD1 knockdown decreased, while overexpression increased, the phosphorylation of PI3K, AKT, and mTOR (**Figure 5B**). These findings indicate that ADGRD1 activates the PI3K/AKT/mTOR signaling pathway both *in vitro* and *in vivo*.

### Inhibition of PI3K/AKT/mTOR signaling reverses ADGRD1-mediated malignant phenotypes

**Figure 6:**
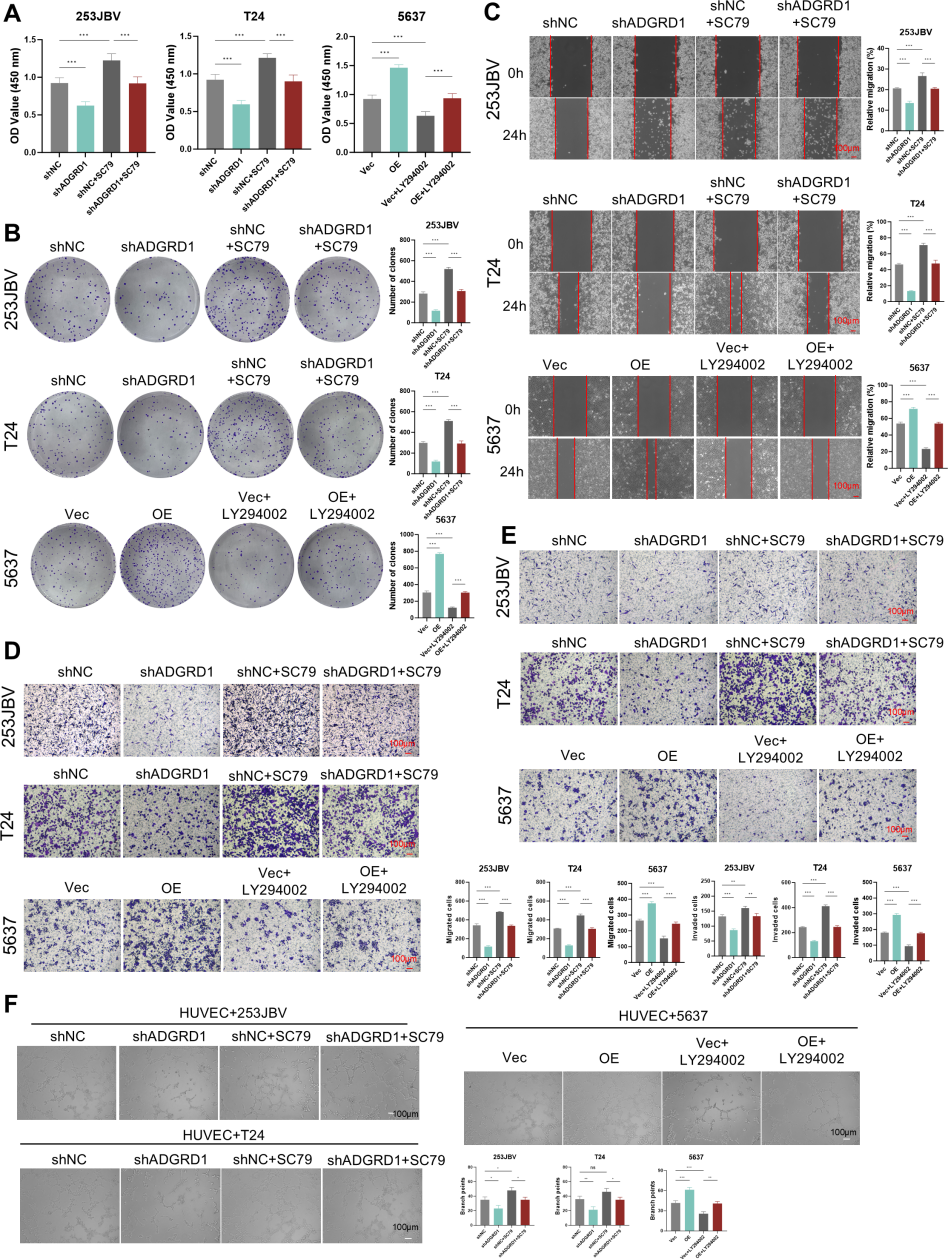
Pharmacological modulation of PI3K/AKT/mTOR signaling reverses ADGRD1-mediated malignant phenotypes.ADGRD1-silenced cells (253JBV, T24) were treated with SC79 (AKT activator), while ADGRD1-overexpressing 5637 cells were treated with LY294002 (PI3K inhibitor). **(A)** CCK-8 assay showing that SC79 rescued, and LY294002 abolished, ADGRD1-dependent changes in cell viability. **(B)** Colony formation, **(C)** wound-healing, **(D)** Transwell migration, **(E)** Transwell invasion, and **(F)** HUVEC tube formation assays demonstrating consistent reversal of ADGRD1 effects upon pathway modulation. Scale bars, 100 µm. Data are presented as mean ± SD of three independent biological replicates. *P < 0.05, **P < 0.01, ***P < 0.001.

To further confirm the role of PI3K/AKT/mTOR signaling in ADGRD1-mediated oncogenic effects, pharmacological intervention experiments were conducted. Treatment with the pathway agonist SC79 rescued the inhibitory effects of ADGRD1 knockdown on cell proliferation, migration, invasion, and angiogenesis in 253JBV and T24 cells. Conversely, the PI3K/AKT/mTOR inhibitor LY294002 abrogated the pro-tumorigenic effects of ADGRD1 overexpression in 5637 cells (**Figures 6A–F**).

These results confirm that the PI3K/AKT/mTOR signaling axis is a key downstream effector required for ADGRD1-mediated oncogenic and pro-angiogenic effects in BLCA.

## Discussion

In this study, we identified ADGRD1 as a novel oncogenic driver in bladder cancer (BLCA) that promotes tumor progression and angiogenesis through activation of the PI3K/AKT/mTOR signaling pathway. Our integrative analysis combining public datasets, *in vitro* assays, and *in vivo* xenograft models provides compelling evidence that ADGRD1 overexpression correlates with aggressive tumor phenotypes and poor clinical outcomes. Mechanistically, ADGRD1 enhances tumor cell proliferation, migration, and invasion, while also stimulating endothelial cell-mediated angiogenesis via the upregulation of a pro-angiogenic secretome. Importantly, pharmacological inhibition of PI3K/AKT/mTOR signaling axis effectively reversed these malignant effects, underscoring its pivotal role in ADGRD1-mediated oncogenic activity.

ADGRD1 (also known as GPR133) belongs to the adhesion G-protein-coupled receptor (aGPCR) family, which is increasingly recognized for its involvement in tumorigenesis and metastasis (11, 13). Prior studies have shown that several aGPCR members, such as ADGRG1 (GPR56) and ADGRL4 (ELTD1), are implicated in cancer progression, extracellular matrix interaction, and angiogenesis (14–18). However, the biological function of ADGRD1 in urologic malignancies has remained largely unexplored. Our data demonstrate for the first time that ADGRD1 expression is markedly elevated in advanced-stage BLCA, consistent with findings in glioblastoma and lung adenocarcinoma where ADGRD1 activation promotes tumor growth and hypoxia adaptation (11, 19, 20).

An important observation from our study is that ADGRD1 does not exhibit uniform overexpression in BLCA tissues compared with normal urothelium across all public datasets. Instead, its expression appears to be context-dependent and associated with tumor progression rather than tumor initiation. This pattern is consistent with increasing evidence that many oncogenic regulators, particularly adhesion GPCRs, display heterogeneous expression across tumor types and stages while exerting strong functional effects in specific biological contexts (6, 11, 21). Our findings suggest that ADGRD1 may be preferentially upregulated in aggressive or metastatic subpopulations of BLCA, where it contributes to proliferation, invasion, and angiogenesis via activation of the PI3K/AKT/mTOR pathway. Therefore, its clinical relevance may lie more in disease progression and prognosis rather than early tumor detection. Clinically, our analysis using GEPIA3 platform confirms that high ADGRD1 expression is a robust predictor of poor prognosis, showing a significant correlation with reduced overall survival (HR = 1.59) and progression-free interval (HR = 1.46).

Our results demonstrate that ADGRD1 exerts strong pro-proliferative and pro-migratory effects in BLCA cells. Silencing ADGRD1 markedly suppressed cellular viability and colony formation, whereas its overexpression yielded the opposite effect. This is consistent with prior observations linking aGPCR activation to enhanced cell survival and motility (6, 7, 22). Mechanistically, we observed that ADGRD1 modulates phosphorylation of PI3K, AKT, and mTOR—key regulators of cell growth, metabolism, and cytoskeletal remodeling (23, 24). Pharmacological rescue experiments using SC79 (AKT agonist) and LY294002 (PI3K inhibitor) confirmed that PI3K/AKT/mTOR signaling is a functional requirement for ADGRD1-driven malignant behaviors, establishing a causal relationship between ADGRD1 expression and downstream oncogenic signaling.

Angiogenesis is essential for bladder cancer progression and is driven by both intrinsic tumor signals and paracrine communication with endothelial cells. Our HUVEC tube formation assays using conditioned medium (CM) revealed that ADGRD1-high BLCA cells secrete factors that enhance vascularization and increase endothelial CD31/CD34 expression. Crucially, we identified that ADGRD1 regulates the mRNA expression of a potent angiogenic “cocktail” consisting of VEGF, PDGF, and IL-8. Similar paracrine mechanisms have been reported for aGPCRs such as ELTD1, which enhances endothelial angiogenesis via Wnt signaling (25). By driving the production of VEGF, PDGF, and IL-8, ADGRD1 essentially ‘reprograms’ the tumor secretome to favor endothelial cell activation, evidenced by the increased expression of CD31 and CD34 in xenograft tumors. This indirect regulatory mode explains the strong clinical correlation we observed between ADGRD1 and vascular density in patient datasets. These insights move our findings beyond phenomenological observation and establish a robust causal link between ADGRD1 activity and the recruitment of the tumor vasculature.

Despite these insights, several limitations should be acknowledged in the current study. First, the absence of a non-neoplastic urothelial control, such as SV-HUC-1, limits the direct evaluation of ADGRD1 expression relative to normal bladder epithelium. Importantly, analysis of multiple public datasets in this study indicates that ADGRD1 does not exhibit consistent upregulation in bladder cancer tissues compared with normal urothelium. Instead, our stratified analysis of the TCGA cohort demonstrates that ADGRD1 expression is significantly elevated in advanced-stage tumors compared with early-stage lesions. This suggests that while ADGRD1 may not be a driver of initial transformation, it is a critical ‘progression switch’ that facilitates the transition to a more lethal, metastatic state. Future studies comparing ADGRD1 activity in normal urothelium under different mechanical stresses would further clarify its baseline physiological role.

Second, although our functional experiments demonstrate consistent oncogenic effects of ADGRD1 across multiple *in vitro* and *in vivo* models, bidirectional genetic manipulation was not performed within each individual cell line, which may introduce potential cell line–specific bias. In this study, loss-of-function experiments were conducted in high-ADGRD1-expressing, invasive cell lines (T24 and 253JBV), while gain-of-function experiments were performed in a low-expressing cell line (5637), to preserve biological relevance and avoid non-physiological overexpression artifacts. Notably, the consistency of phenotypic outcomes across different cell lines, together with *in vivo* xenograft validation and pharmacological pathway modulation, supports the robustness of our conclusions. Nevertheless, future studies incorporating paired gain- and loss-of-function approaches within the same cellular background will further strengthen these findings.

Third, the precise receptor-proximal signaling mechanisms underlying ADGRD1-mediated activation of the PI3K/AKT/mTOR pathway remain to be fully elucidated. Adhesion GPCRs like ADGRD1 often utilize a ‘Stachel’ mediated tethered agonist mechanism to initiate signaling (11, 26). In glioblastoma, ADGRD1 has been shown to signal via Gα_s_ to increase intracellular cAMP (10). However, our results in BLCA highlight a robust modulation of the PI3K/AKT/mTOR axis. This suggests that in the urothelial context, ADGRD1 might engage alternative transducers, such as Gα_q_ or β-arrestin-dependent scaffolds, both of which are known to coverage on PI3K activation (27). While our pharmacological establish a clear functional requirement for this cascade, further studies employing BRET-based biosensors or G-protein specific inhibitors are warranted to map the direct physical coupling of ADGRD1 in the bladder. Additionally, whether ADGRD1 modulates endothelial behavior directly through these secreted cytokines or indirectly via exosomes warrants further exploration.

## Conclusion

In summary, our findings identify ADGRD1 as a progression-associated oncogenic regulator in bladder cancer that promotes tumor growth and angiogenesis via the modulation of the PI3K/AKT/mTOR pathway. Targeting ADGRD1 or its downstream signaling may provide a new therapeutic avenue for treating advanced BLCA. These results extend the understanding of adhesion GPCRs in tumor biology and establish a rationale for further translational investigation of ADGRD1 in cancer therapy.

## Data Availability

The raw data supporting the conclusions of this article will be made available by the authors, without undue reservation.
