# Supplementary figures and images for: ADGRD1 promotes bladder cancer progression and angiogenesis via the PI3K/AKT/mTOR-mediated pro-angiogenic secretome
Source: Front Oncol. 2026 May 28;16:1817872. doi: 10.3389/fonc.2026.1817872 (PMC13253281; doi:10.3389/fonc.2026.1817872)

Figure 1D

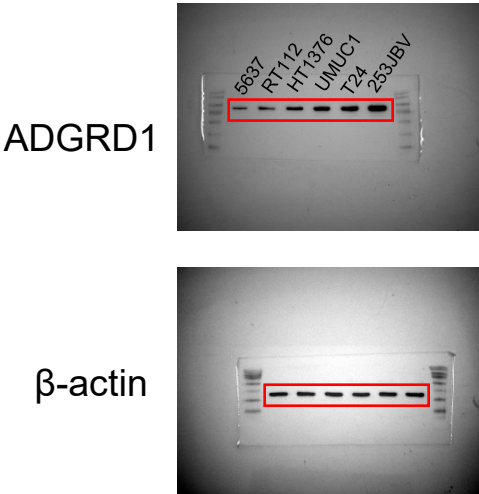

Figure 2B

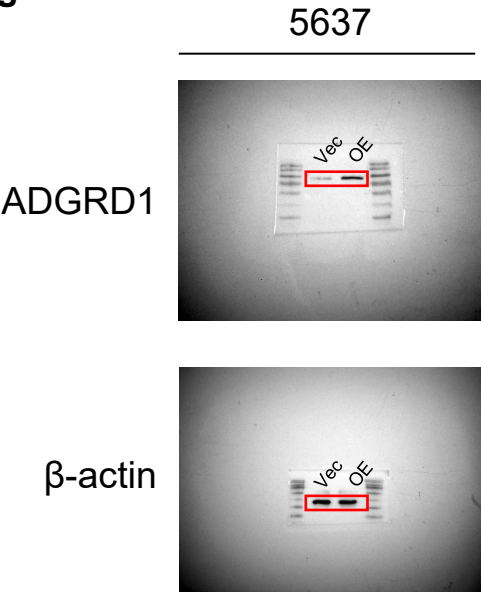

Figure 2B

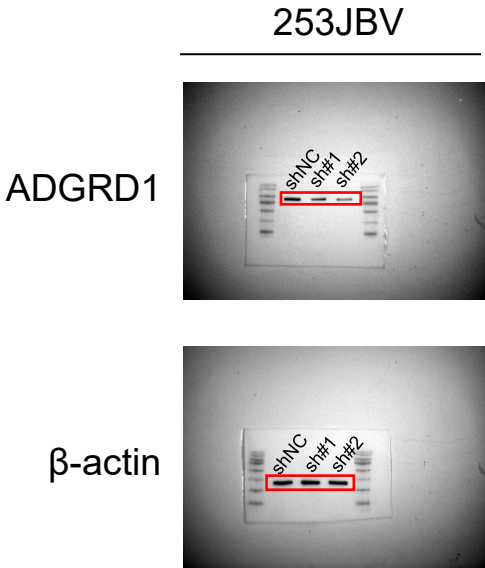

Figure 3C

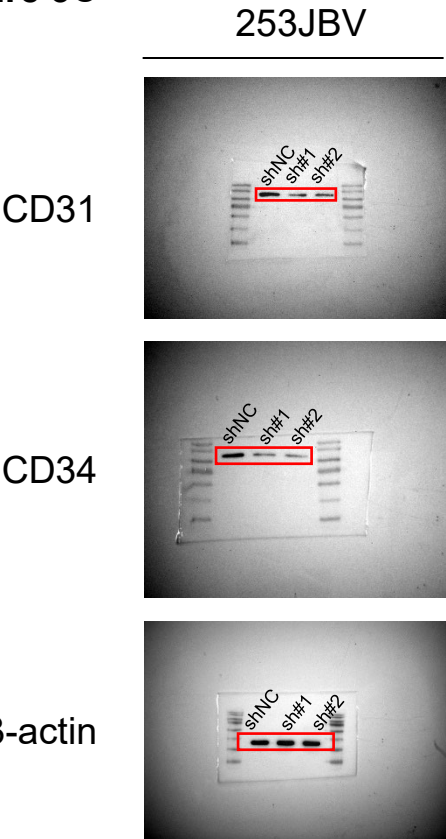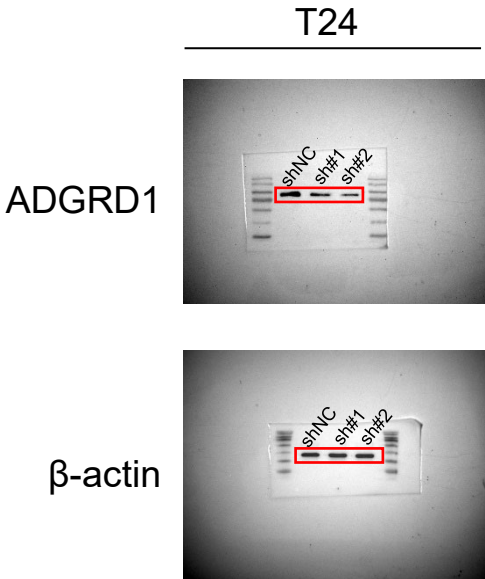

Figure 3C

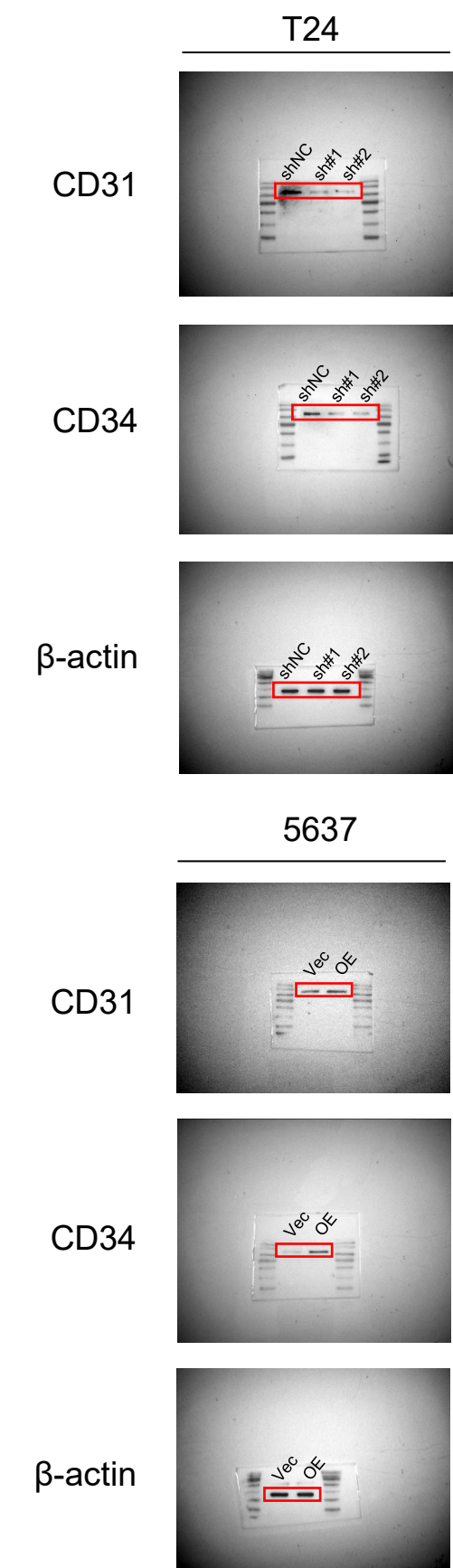

Figure 5A

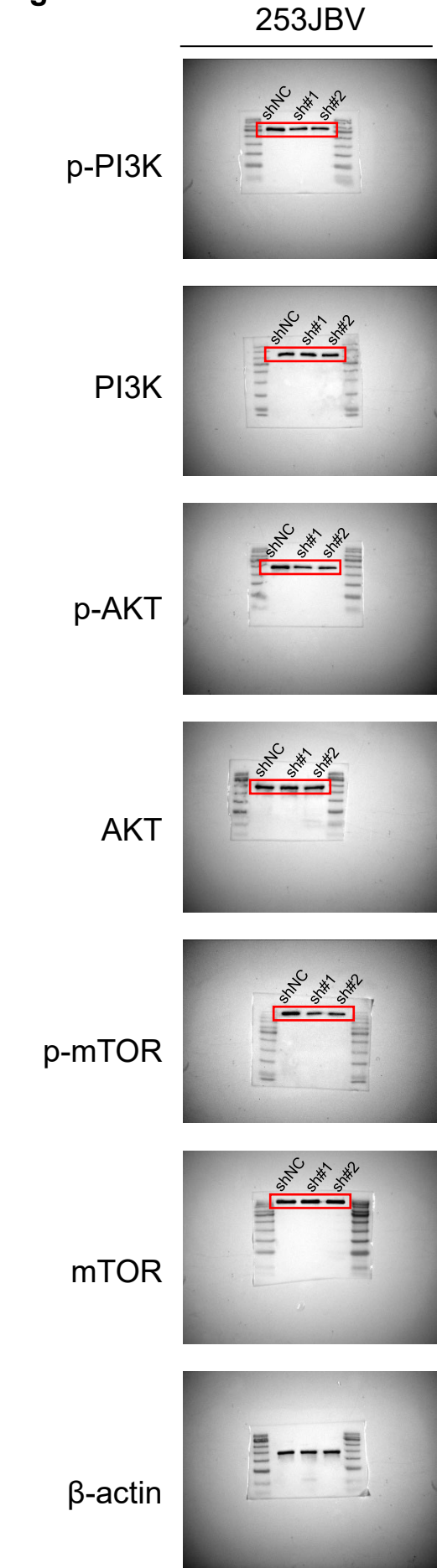

Figure 5A

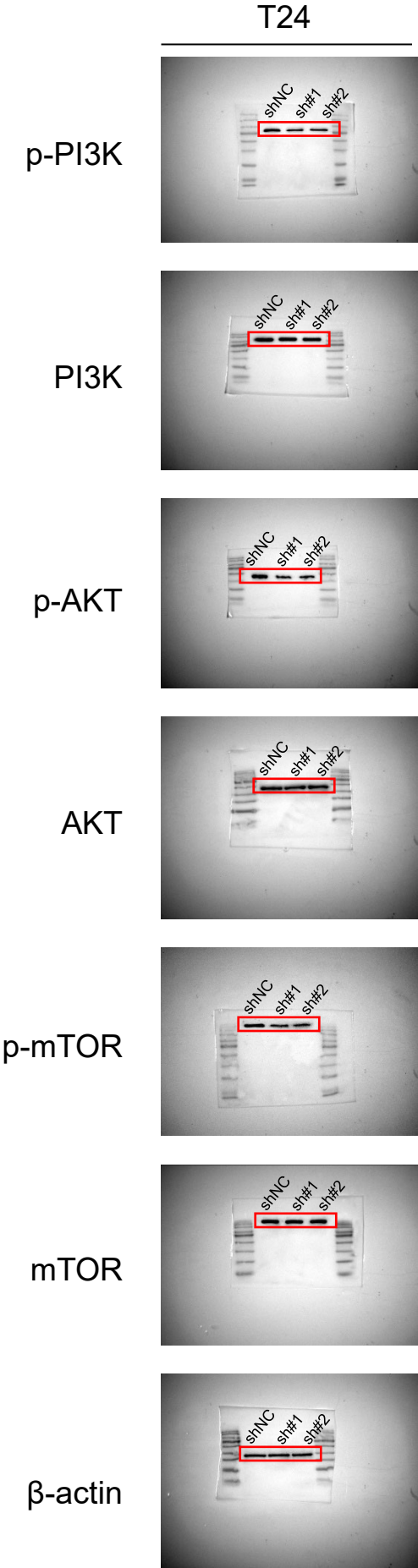

Figure 5A

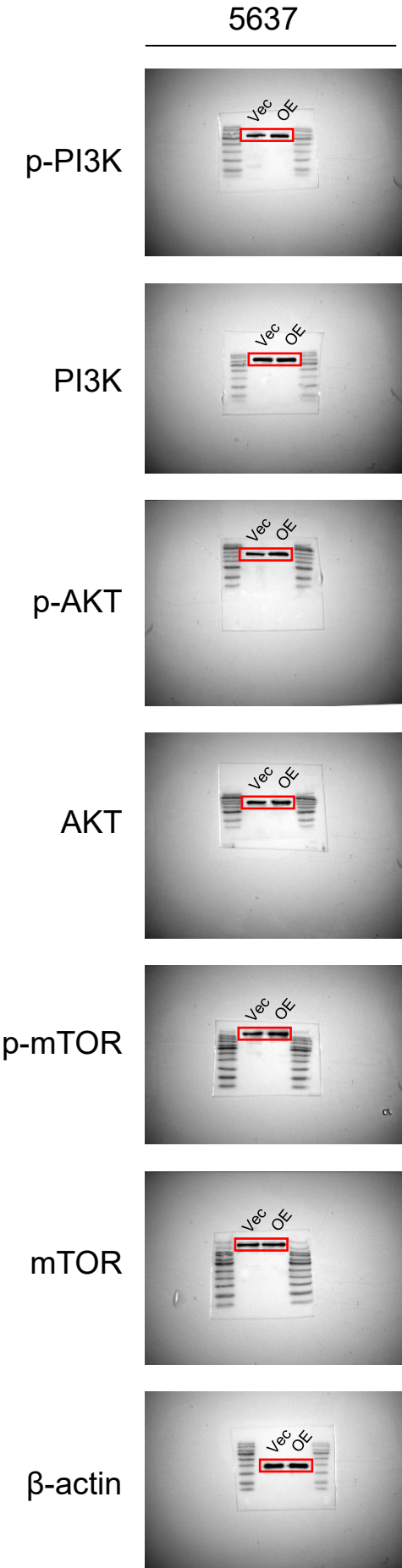

Figure 5B

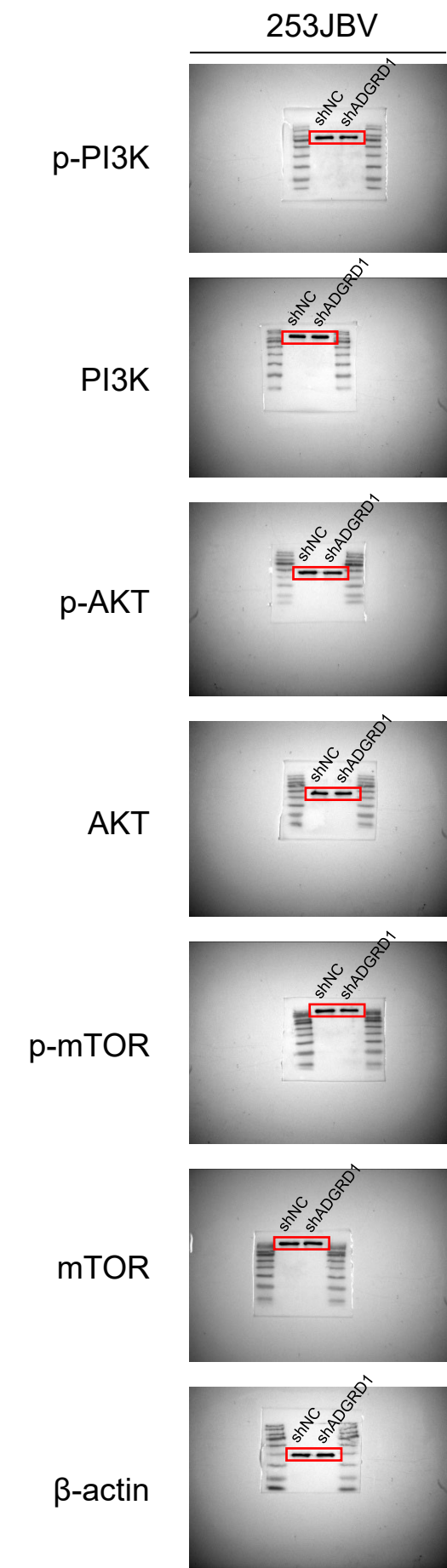

Figure 5B

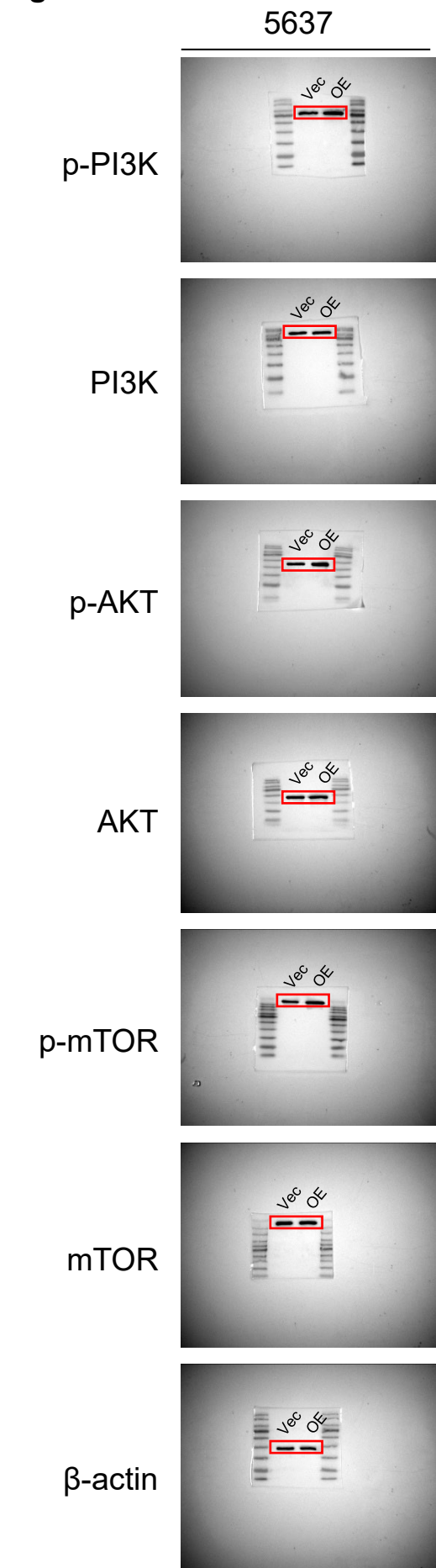

Supplement: Supplementary file 1 [file DataSheet1.pdf]
