# Supplementary material for: ADGRD1 promotes bladder cancer progression and angiogenesis via the PI3K/AKT/mTOR-mediated pro-angiogenic secretome
Source: Front Oncol. 2026 May 28;16:1817872. doi: 10.3389/fonc.2026.1817872 (PMC13253281; doi:10.3389/fonc.2026.1817872)

**Supplementary figure S1. Cross-dataset analysis of ADGRD1 expression in bladder cancer.**

(A) Analysis of ADGRD1 mRNA expression in bladder cancer (BLCA) tissues versus normal urothelial tissues using the TCGA-BLCA cohort via the GEPIA3 platform. Data are presented as log2(TPM + 1).

(B) Validation of ADGRD1 expression across independent Gene Expression Omnibus (GEO) datasets, including GSE7476 (B), GSE40355 (C), GSE121711 (D), and GSE293398 (E), comparing tumor and normal bladder tissue samples.

(C) Stratified analysis of ADGRD1 expression within the TCGA-BLCA cohort showing significantly higher expression in advanced-stage tumors compared with early-stage tumors, supporting a **progression-associated expression pattern.**


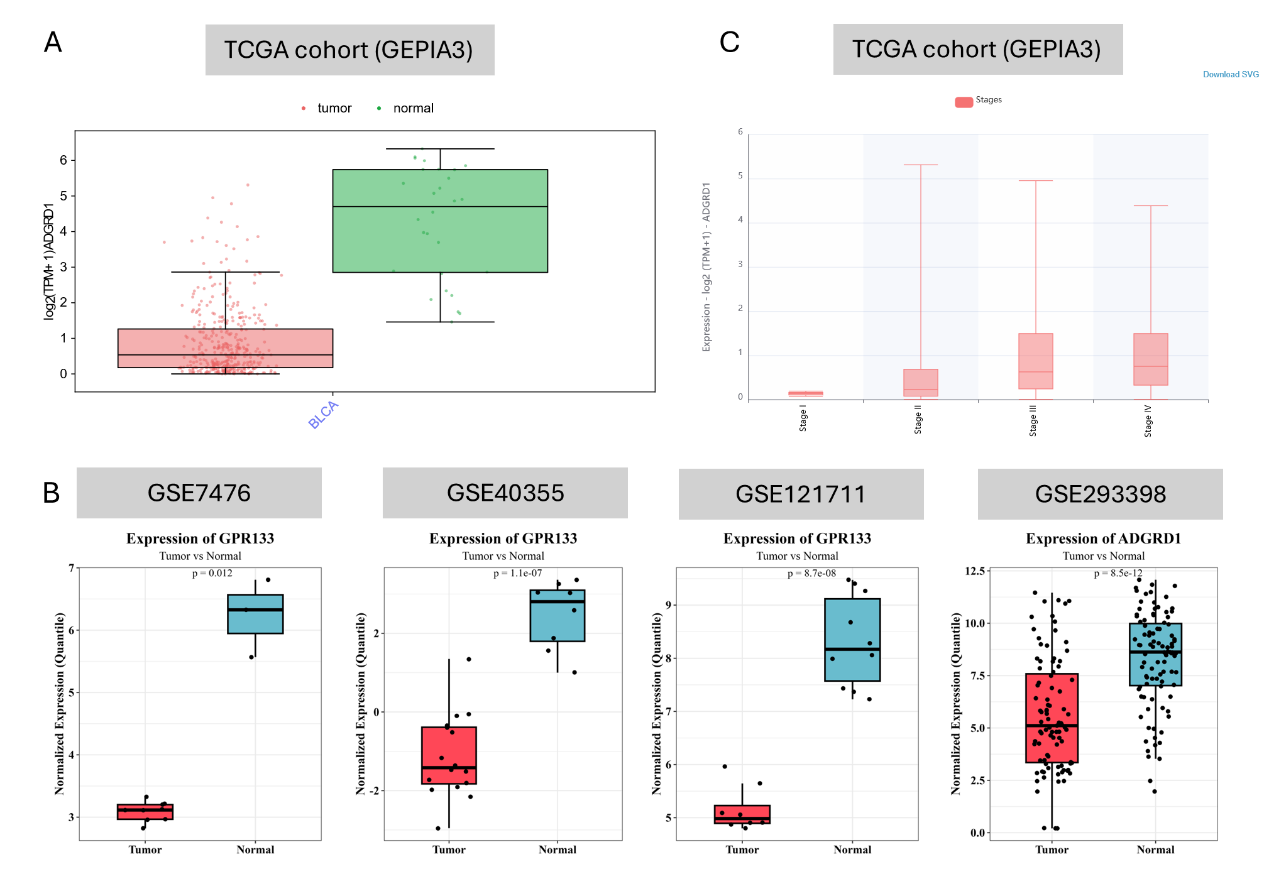

Supplement: Supplementary file 2 [file DataSheet2.docx]
